# Editorial perspective: Facilitating access to mental health research participation for children in care: Lessons from the ReThink project

**DOI:** 10.1002/jcv2.70114

**Published:** 2026-03-25

**Authors:** Charlotte Robinson, Katherine H. Shelton, Lisa Holmes, Bethan Carter, Eva A. Sprecher, Ace Garmadon, Clo H, Rachel M. Hiller

**Affiliations:** ^1^ School of Psychology Cardiff University Cardiff UK; ^2^ School of Education and Social Work University of Sussex Brighton UK; ^3^ Clinical, Educational & Health Psychology University College London London UK

**Keywords:** care‐experienced, foster care, methods, out‐of‐home care, recruitment

## Abstract

Children in care have historically been under‐represented in mental health research, despite high levels of need. Consequently, there is a lack of high‐quality empirical evidence to drive advocacy, practise, and policy, and the direct voice of children in care is often absent. In this Editorial Perspective, we outline three key areas of consideration, that must be understood and addressed to maximise the success of primary mental health research with this group of children. Specifically, we focus on: capacity issues in children's social care and partnership working; consent and assent procedures; and supporting children in care through mental health research. The paper is informed by the ReThink Project, a longitudinal mixed‐methods study involving 450 care‐experienced young people across 13 local authorities in England and Wales. The issues and solutions we discuss have implications for future study design, including timelines and funding.

## Key Points

**What's known?**

- Children in care have historically been excluded from and under‐represented in mental health research, particularly when considering their level of need.
- There is a lack of high‐quality empirical evidence, and the direct voice of children in care is often absent.

**What's new?**

- We examine key challenges that have constrained primary data collection on the mental health of children in care and propose solutions informed by our experience on a large, longitudinal mixed‐method study (The ReThink Programme).

**What's relevant?**

- The issues and solutions we discuss are important for other research projects to consider when embarking on primary data research on the mental health and wellbeing needs of children in care.
- Implications for future study design, including timelines and funding.

## Graphical abstract

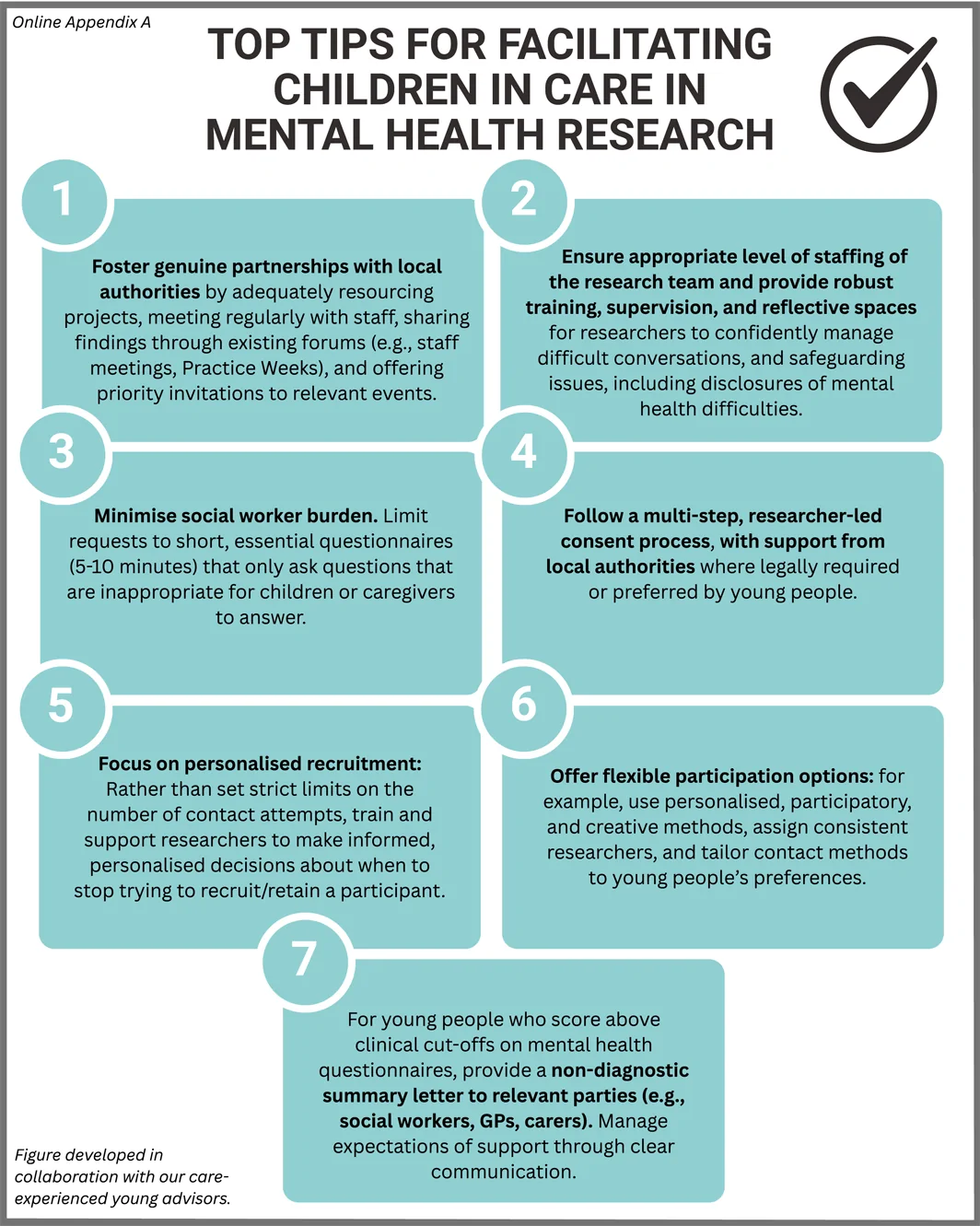

## INTRODUCTION

Children in care have historically been excluded from and under‐represented in mental health research, particularly when considering their level of need (Children's Commissioner & Coram, 2023; Fledderjohann et al., 2021; Ruff et al., 2025). Existing research tends to be small scale and under‐powered or relies on administrative data which can lack depth. Consequently, there is a lack of high‐quality empirical evidence to drive advocacy, practise, and policy decision‐making, and the direct voice of children in care is often absent.

This paper examines three key challenges that have constrained robust primary data collection on the mental health of children in care; we also propose solutions informed by our experience on the ReThink Project. These challenges are: (i) limited capacity within children's services to support research; (ii) complexities in consenting procedures; and (iii) appropriate support for children in care during research participation.

**FIGURE 1:**
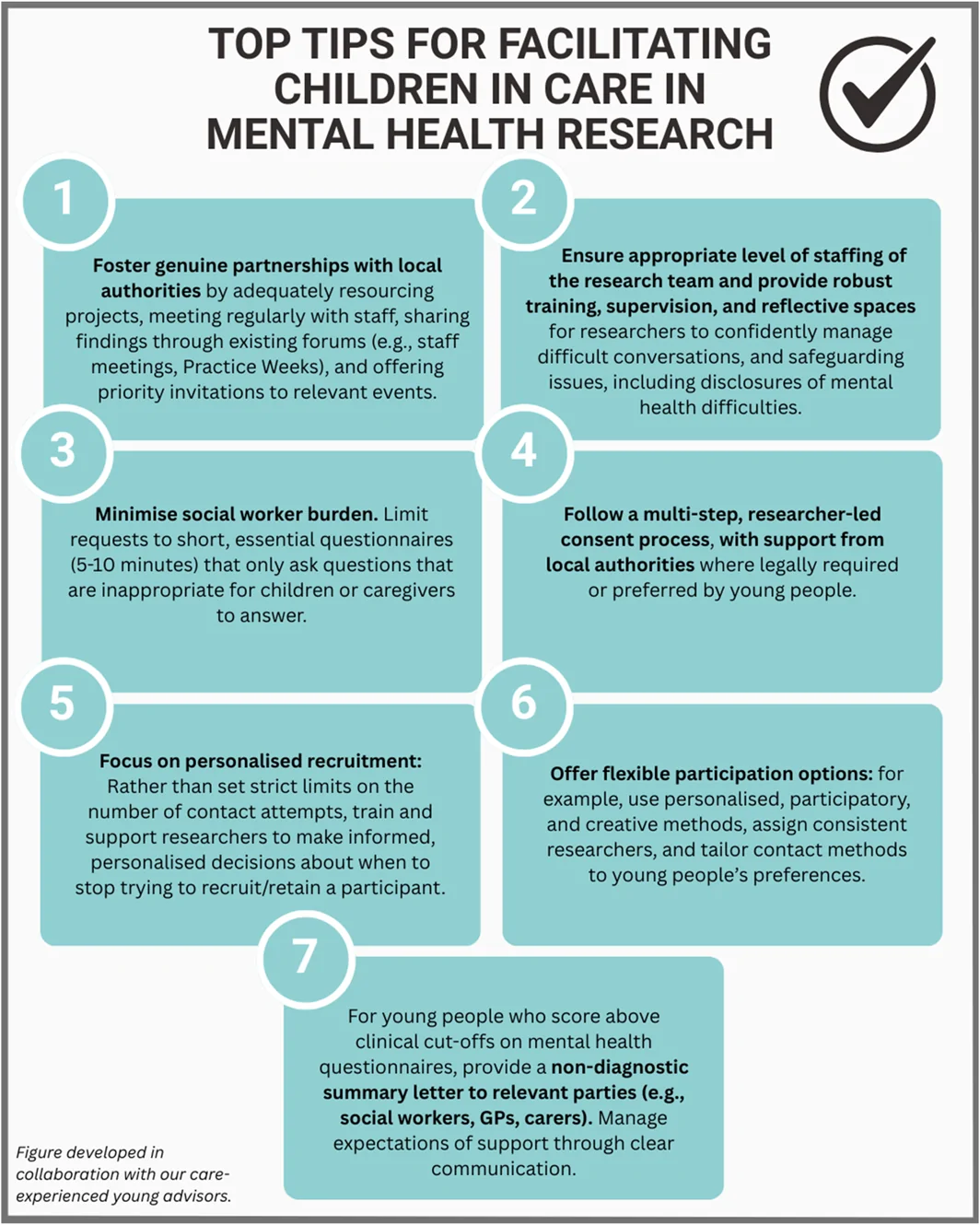
Top tips for facilitating children in care in mental health research.

The ReThink Project (Hiller et al., 2023 [https://osf.io/7qx54/overview]) was a large research programme exploring drivers of mental health and wellbeing in care‐experienced young people. The overall programme was supported by a multidisciplinary team of academics, two national charities, and care‐experienced advisory boards. The programme included a primary data longitudinal mixed‐methods work package, which investigated psychological, social, and service‐level factors influencing the mental health and wellbeing of care‐experienced young people during important life transitions (moving to secondary school; leaving care and entering adulthood). This work package predominantly recruited children and young people in care (i.e., currently under local authority care), but also included some children who had been adopted from care (i.e., care‐experienced children). The final sample included 450 care‐experienced children (288 aged 10–13; 162 aged 16–17), along with caregivers. Retention at 1‐year follow‐up was approximately 73%, and social worker‐reported data were completed for over 90% of participants. The current paper focuses on the recruitment of those in the care system, which was 92% of our sample, and presents key strategies and considerations to support large primary research with this population. Figure 1 provides a visual summary of our key strategies, developed in collaboration with care‐experienced young advisors.

## LOCAL AUTHORITY CAPACITY AND THE NEED FOR A CORE PRINCIPLE OF MINIMAL BURDEN

Local authority children's services act as the primary gatekeepers for recruiting children in care. With limited exceptions, recruiting these young people (particularly if under 16 years) cannot happen without formal approvals from local authority professionals. Children's services departments are incredibly busy, often under‐resourced and have high staff turnover from the frontline to leadership level (Baginsky, 2023; Johnson et al., 2023); these services often do not have established research structures like the NHS (although this is improving). It is essential for researchers to work in genuine partnership with local authorities, recognising what is feasible, troubleshooting where possible, respecting the professional expertise of social care staff, and engaging local authorities as stakeholders in achieving meaningful outcomes. This requires the following considerations:

### Resourcing and timelines

Projects should be planned and resourced to adequately reflect the work needed to build and maintain relationships with local authorities. It took between 3 and 12 months to set‐up each local authority site ready for recruitment (sometimes this included developing new research governance processes). The ReThink project partnered with 13 local authorities across England and Wales; recruitment of the 450 young people took 24 months; and this was supported by two full‐time postdoctoral researchers and two research assistants, along with the senior academic team. Not all projects will need this level of staffing, of course, but this is an example of the resources required for longitudinal projects.

### Project development with local authority buy‐in from the outset and ongoing feedback loops

Project development should be recognised as a crucial aspect of co‐production, helping to ensure that the research is viewed as a priority by services and therefore worthy of their limited time. To maintain engagement and show genuine appreciation, the ReThink team regularly met with local authority staff, shared findings (project and broader work) at appropriate points through forums such as staff meetings and Practise Weeks and offered priority invitations to relevant webinars and events.

### Prioritising relationship building and reducing siloed working

Local authorities receive numerous research requests (one site had 10 in a week). Researchers should coordinate across teams to ensure projects complement rather than compete and recruit only from the number of authorities needed for their sample. Spreading recruitment thinly across many authorities is inefficient and burdensome. Focusing on fewer sites supports stronger relationships and clearer understanding of roles. In ReThink, each site had a designated contact, whilst administrative staff handled key tasks instead of frontline social workers; this streamlined communication and reduced workload.

### Minimising social worker burden

We asked social workers to complete a single 5–10‐min questionnaire about the young people on their caseload who were in the project. Questionnaires were completed by over 90% of social workers. Success rested on the principles set out above combined with troubleshooting with managers or site contacts to overcome barriers to completion.

## CONSENT: BALANCING LEGAL REQUIREMENTS WITH THE RIGHTS OF CHILDREN

Obtaining consent is a barrier to carrying out research with children in care, but it is a barrier that can be overcome. It involves a careful balance between the legal responsibility of the local authority and the rights of the child to be involved in research about their own lives. Local authorities hold either full or shared parental responsibility for a child in care, but in many cases the person who would legally sign the consent form might barely know the child. The caregiver the child lives with is often (not always) the best placed adult to consider whether the project is suitable for the child but does not hold parental responsibility to consent. Delegated authority (allowing foster carers to make certain decisions for the child) is also applied differently between local authorities. Depending on the legal order, some children may also require consent (or at least an opportunity to opt‐out) from the biological parent.

### Multi‐step consent process

In the ReThink Project we had a multi‐step process, which ensured local authorities provided the necessary consent for potentially eligible children, but were not unduly gate‐keeping children from deciding whether they wished to participate. Crucially, processes were co‐developed with services and children. Our process was supported by an agreement that the research team would be responsible for talking to carers and children about the project. In earlier studies where social workers handled consent, limited capacity and inaccurate information often disrupted procedures and increased burden. We also saw cases where social workers assumed a child would decline, only for the child to participate enthusiastically. Much of the success of our consenting process stemmed from our commitment to full partnership working.

Steps included opt‐out letters sent from local authorities; local authority consent to allow children to participate *if they want to*; secure sharing of minimal contact details; discussion with caregivers where feasible (even if they were not providing consent), and fully informed young person assent. Sixteen‐ and 17‐year‐olds provided their own informed consent. Gillick competency should be carefully considered, where appropriate. In any case, it is important for the local authority to be involved in some capacity to facilitate appropriate safeguarding.

### Challenges with contacting young people and caregivers, and the need for robust training and supervision of researchers to manage conversations

The process was not without its challenges, particularly given capacity issues, staff turnover, and errors in service data. Lags in the timeline to receive contact details meant some children had become ineligible by the time they were contacted by the research team (i.e., were no longer in the age range); 24% of children were not contactable using the details provided (e.g., contact details were not for the current caregiver, there were missing telephone numbers, or telephone numbers were inactive); 26% were discovered to not be eligible after initial contact (e.g., did not have adequate English or intellectual ability). It was often not clear who the contact details were for children living in residential care (e.g., a key worker, a residential care home manager). That these issues were often only discovered after the research team made initial contact meant it was crucial that the team received training and supervision from experienced researchers in how to manage these conversations sensitively. Our co‐production with children, young people and services also helped us navigate this. Logistical timeline challenges like these often required going back to sites, reporting issues and sometimes obtaining new details.

In addition to robust training and supervision of junior researchers, considerable flexibility was needed. Often, contacting the carer and child took multiple attempts, even when children and carers expressed initial interest in the project. Sometimes, even when contact was made, participation was postponed because the child was experiencing a difficult period (e.g., distress, hospital admission, bereavement, placement move). We would discourage set rules about how many phone calls to make and instead focus on training and supporting researchers to make personalised and informed decisions about when to cease trying to recruit a participant. Additionally, even initial straight forward recruitment calls could take 30 min or more, as caregivers or children often opened up about their experiences. Again, this speaks to the need for robust training and supervision for team members.

## SUPPORTING CHILDREN WITH THE RESEARCH PROCESS AND RETENTION IN LONGITUDINAL RESEARCH

Children in care often report feeling that assessments are done *on* them and not *with* them ‐ something that we were committed to not repeating or reinforcing in ReThink. When researching with children in care, particularly mental health research, it is crucial that the full team are trained to sensitively and effectively manage wellbeing and safeguarding issues. This includes ensuring young people are aware that any safeguarding concerns will be shared in line with safeguarding procedures. Engagement must also ensure that children's participation feels supported and meaningful.

We approached this research anticipating high rates of mental health needs, dissatisfaction with support across mental health, education, and social care settings, and challenges such as interpersonal difficulties, placement instability, and self‐harm. Many children we worked with had never engaged in in‐depth conversations about their mental health before participating in the study. This responsibility must be taken seriously by researchers. Our work was guided by the following principles:

### Involvement of children in care from the outset

Wellbeing and safeguarding procedures, information sheets, and study materials were co‐developed with input from care‐experienced young people, including those currently in care, and with services. This led to practical decisions that supported engagement, such as offering flexible questionnaire options (online, in‐person, with or without support), minor amendments to language in standardised questionnaires (e.g., 'parent' to 'carer'), using participatory and creative methods, assigning consistent researchers, avoiding automated emails in favour of personalised communication, and tailoring contact to participant preferences. These steps offered reassurance that participation would be handled with care and emphasised that the research aimed to provide supportive, meaningful experiences for participants.

### Prioritisation of wellbeing and safeguarding training for the research team, with ongoing reinforcement through structured reflective sessions and regular professional supervision

Interpersonal issues, self‐harm and suicidal ideations, and unstable living situations, are all issues that are more common amongst children in care. Researchers must approach their work with the appropriate resources, training and expectation that they will need to be able to confidently manage disclosures of mental health difficulties and safeguarding or risk concerns. Senior researchers should also recognise the impact this can have on junior team members, and strong supervision structures are vital. On ReThink, we had weekly full team meetings; the team also held weekly reflective spaces that the senior team did not attend; and we encouraged an open environment where researchers could discuss how conversations might have affected them.

### Assessment summaries provided as standard, where concerns were flagged

For many children, this was their first experience completing comprehensive mental health questionnaires. Many scored above clinical cut‐offs. When this happened, we provided a summary to the social worker to support potential referrals, which was discussed openly with children and caregivers coupled with clear communication that this did not guarantee support. For older participants, summaries could be sent directly to them or their GP, which was important for those without a consistent caregiver. Language was carefully chosen to ensure it was clear that this was a research project (not clinical assessment) and to clarify that assessments were not diagnostic. This approach also demonstrated to local authorities that we took our responsibility toward children seriously.

## CONCLUSION

There is an urgent need for larger‐scale powered primary data research on the mental health and wellbeing needs of children in care, particularly that which moves beyond describing the problem to identifying potential solutions. This paper has summarised, and illustrated using the ReThink Project, three key areas for other research projects to consider when embarking on this kind of research. These issues have implications for project timelines and funding that should be contemplated ahead of commencing research.

## AUTHOR CONTRIBUTIONS

**Charlotte Robinson**: Data curation; investigation; methodology; project administration; writing—original draft; writing—review and editing. **Katherine H. Shelton**: Conceptualization; formal analysis; funding acquisition; investigation; methodology; supervision; writing—original draft; writing—review and editing. **Lisa Holmes**: Conceptualization; formal analysis; funding acquisition; investigation; methodology; project administration; supervision; writing—original draft; writing—review and editing. **Bethan Carter**: Conceptualization; data curation; formal analysis; investigation; methodology; project administration; writing—original draft; writing—review and editing. **Eva A. Sprecher**: Conceptualization; data curation; formal analysis; investigation; methodology; project administration; writing—original draft; writing—review and editing. **Ace Garmadon.** Methodology; visualization; writing—review and editing. **Clo H**: Methodology; visualization; writing—review and editing. **Rachel M. Hiller**: Conceptualization; formal analysis; funding acquisition; investigation; methodology; writing—original draft; writing—review and editing.

## CONFLICT OF INTEREST STATEMENT

The authors declare no conflicts of interest.

## ETHICAL CONSIDERATIONS

Ethical approval was granted by the University College London Research Ethics Committee (23/02/2022; Ref: 22253/001), alongside approval from the Association of Directors of Children's Services and the research governance processes of participating local authorities. Informed consent for young people's participation in the ReThink Project was obtained from senior social care professionals, with caregivers and young people providing consent or assent as appropriate. Safeguarding and risk escalation protocols were implemented, and mental health assessment summaries were shared with social workers or (adoptive) parents where required.

## Data Availability

Data sharing not applicable to this article as no datasets were generated or analysed during the current study.

## References

[jcv270114-bib-0001] Baginsky, M. (2023). Parents’ views on improving relationships with their social workers. Journal of Social Work, 23(1), 3–18. 10.1177/14680173221101244

[jcv270114-bib-0002] Children’s Commissioner & Coram . (2023). Findings from the big ask: Children in care. Children's Commissioner for England. Retrieved from https://www.childrenscommissioner.gov.uk/resource/findings‐from‐the‐big‐ask‐children‐in‐care/

[jcv270114-bib-0003] Fledderjohann, J. , Erlam, J. , Knowles, B. , & Broadhurst, K. (2021). Mental health and care needs of British children and young people aged 6–17. Children and Youth Services Review, 126, 106033. 10.1016/j.childyouth.2021.106033

[jcv270114-bib-0004] Hiller, R. , Holmes, L. , Carter, B. , Sprecher, E. A. , Macleod, J. , Selwyn, J. , Shelton, K. , & Siraj, I. (2023). The ReThink programme work package 2: A longitudinal investigation of the mental health and wellbeing of care‐experienced young people [preregistration]. Open Science Framework. Retrieved from https://osf.io/7qx54/overview

[jcv270114-bib-0005] Johnson, C. , Jouahri, S. , Earl, S. , White, Y. , Woods, D. , Pollock, S. , Scholar, H. , & McCaughan, S. (2023). Longitudinal study of local authority child and family social workers (wave 5) (Research report). Department for Education. Retrieved from https://assets.publishing.service.gov.uk/government/uploads/system/uploads/attachment_data/file/1170189/Longitudinal_study_of_local_authority_child_and_family_social_workers_Wave_5.pdf.pdf

[jcv270114-bib-0006] Ruff, S. , Linville, D. , Ramirez, C. , Vasquez, N. , & Schwabenland, C. (2025). A qualitative investigation of foster youth mental health outcomes: Measuring what matters. Journal of Child and Family Studies, 34(3), 587–600. 10.1007/s10826-025-03035-w

